# The effect of regular aerobic exercise on renal function in patients with CKD: A systematic review and meta-analysis

**DOI:** 10.3389/fphys.2022.901164

**Published:** 2022-09-26

**Authors:** Qirui Ma, Ye Gao, Jiandong Lu, Xinhong Liu, Ruolin Wang, Yajun Shi, Jingqi Liu, Hao Su

**Affiliations:** ^1^ The Graduate School, Beijing Sport University, Beijing, China; ^2^ The School of Sports Science, Beijing Sport University, Beijing, China

**Keywords:** aerobic exercise, chronic kidney disease, kidney function, meta-analysis, systematic review

## Abstract

**Objective:** To evaluate the effect of regular aerobic exercise on the improvement of renal function in patients with chronic kidney disease through meta-analysis and to provide targeted exercise recommendations for patients with CKD.

**Methods:** PubMed, Web of Science, EBSCO, China National Knowledge Infrastructure (CNKI), and other databases were searched, and randomized controlled trials on the effects of regular aerobic exercise on renal function-related indexes in patients with CKD were collected according to the inclusion and exclusion criteria. The methodological quality of the included literature was evaluated using the Cochrane evaluation tool second generation, and statistical analysis was performed using R analysis software.

**Results:** A total of 12 randomized controlled trials (RCTs) with a total of patients with CKD were included, and the results of the meta-analysis showed that regular aerobic exercise significantly improved the estimated glomerular filtration rate SMD = 0.65, 95% CI [0.30, 1.00], serum creatinine SMD = -0.63, 95% CI [-0.86, -0.40], 24-h urine protein volume in patients with CKD SMD = -0.41, 95% CI [-0.70, -0.11], and serum urea nitrogen SMD = -0.66, 95% CI [-1.20, -0.12]. Single exercise session longer than 30 min significantly improved the estimated glomerular filtration rate in CKD patients (*p* < 0.01), and walking and running as exercise modalities significantly improved CKD patients’ SCr levels were significantly improved by walking and running as exercise modalities (*p* < 0.05), and the improvement effect was not significant when cycling was selected as an exercise modality.

**Conclusion:** Regular aerobic exercise has a significant effect on the estimated glomerular filtration rate, serum creatinine, 24-h urine protein amount, and blood urea nitrogen in CKD patients. Aerobic exercise with a single exercise duration longer than 30 min has a more significant effect on the estimated glomerular filtration rate, and aerobic exercise by walking or running can more effectively improve the serum creatinine in CKD patients.

## 1 Introduction

Chronic kidney disease (CKD) refers to the structural and functional disorders of the kidney caused by various reasons, usually manifested as a decrease in the glomerular filtration rate (GFR) and a decrease in renal function. At the beginning of the disease, patients only have mild symptoms such as weakness, lumbago, and loss of appetite, while entering in the early stages of the disease, patients only have mild symptoms such as weakness, lumbago, loss of appetite, etc., but after entering CKD stage 3, kidney failure may be accompanied by hypertension, heart failure, hyperkalemia, and other adverse symptoms and may even be life-threatening. Current epidemiological studies show that the incidence of CKD is increasing year by year worldwide, and the mortality rate of the affected population reaches 40 times that of the normal population (Collins et al., 2003), making CKD one of the major diseases threatening public health.

Since the initial symptoms of CKD are not obvious, many patients often miss the critical time for early intervention and control of the disease process after diagnosis, and once they enter the end stage, that is, end-stage renal disease (ESRD), they can only be treated by hemodialysis and kidney transplantation, which is costly and has large side effects on the organism. Therefore, prevention and intervention before or during chronic disease are essential to reduce the prevalence and slow down the disease process, and physical activity is a common non-pharmacological intervention that plays an active role in improving cardiovascular health, increasing aerobic capacity and muscle strength, reducing the inflammatory response, and improving immunity (Leehey, Moinuddin, Bast, Qureshi, & Collins, 2009; Headley et al., 2010; Kosmadakis et al., 2010; Meuwese et al., 2011; Watson et al., 2018). Relevant meta-analysis has shown that the incidence of CKD is lower in people with higher physical activity levels (Kelly et al., 2021), among which aerobic exercise is the most commonly used exercise modality in middle-aged and elderly people, but there is a lack of comprehensive quantitative evaluation studies on the effect of aerobic exercise on renal function intervention in CKD patients. Therefore, this study intends to select randomized controlled trials of aerobic exercise on CKD patients at home and abroad and use meta-analysis to evaluate the studies. Therefore, this study is intended to evaluate the consistency of the results among the studies by using meta-analysis to quantitatively evaluate the effects of different aerobic exercise programs on the improvement of renal function in CKD patients of different ages and to provide a theoretical basis and targeted exercise recommendations for the selection of exercise intervention programs for CKD patients.

## 2 Methods

### 2.1 Search strategy

The English search terms used in this article were “kidney,” “kidney function,” “kidney physiology,” “renal function,” “aerobic training,” “aerobic exercise,” and “endurance training.”

The Chinese search terms used in this article were “kidney,” “renal function,” “aerobic exercise,” “aerobic training,” “endurance training,” and “exercise.”

A combination of subject terms and free terms were used to search each database in combination, and the Chinese databases used were China National Knowledge Infrastructure (CNKI) and Wanfang Data Knowledge Service Platform; English databases included PubMed, Web of Science, and EBSCO to collect all literature on the effect of aerobic exercise on the improvement of kidney function. The search time frame was from the date of database construction to September 2021.

### 2.2 Literature inclusion and exclusion criteria

#### 2.2.1 Literature inclusion criteria

The inclusion criteria of the literature were based on the PICO guidelines for evidence-based medicine, and the criteria developed were as follows: 1) study type: all included studies were randomized controlled trials (RCT); 2) literature type: full-text literature of randomized controlled trials of aerobic exercise as an intervention to improve renal function in patients with CKD at home and abroad, in Chinese and English; 3) study subjects: patients with diagnosed CKD, age ≥18 years, age, gender, race, and nationality were not restricted; 4) outcome indicators, including glomerular filtration rate, serum creatinine, urine protein, and blood urea nitrogen.

### 2.2.2 Exclusion criteria of the literature

The exclusion criteria of the literature were as follows: 1) animal studies; 2) cross-sectional studies; 3) data or articles were incomplete and fruitless after requesting from the authors; 4) non-English and Chinese literature.

### 2.3 Literature screening

Step 1: Import the retrieved literature into the reference management software Endnote. Step 2: Eliminate duplicate literature. Step 3: Read the title and abstract for the first round of screening. Step 4: Download the full text for the second round of screening and determine whether the inclusion criteria are met.

### 2.4 Data extraction

Two authors, Qirui Ma and Gao Ye, extracted the literature data separately, and the data were cross-checked and then included in the analysis process. If the extracted data were different, they were discussed with the third author, Xinhong Liu, and then included. When the literature was incomplete, the original authors were contacted to improve the relevant data.

The extracted data included first author, year of publication, study site, sample size, baseline characteristics of patients (age, male/female ratio, and physical condition), intervention modality (intervention content, intervention period, intervention frequency, single intervention duration, and intervention intensity), and outcome evaluation index.

### 2.5 Literature quality evaluation

The included studies were evaluated using the revised Cochrane risk-of-bias tool for randomized trials (RoB 2) in terms of risk of bias arising from the randomization process, risk of bias due to deviations from the intended interventions (effect of assignment to intervention), risk of bias due to missing outcome data, risk of bias in the measurement of the outcome, risk of bias in the selection of the reported result were assessed in five main parts. Finally, an overall evaluation of the article was performed.

### 2.6 Data analysis

The meta-analysis was performed according to the guidelines for systematic evaluation and meta-analysis (PRISMA). All study data were continuous variables and measured in the same units, so SMD and 95% confidence interval (CI) were used for statistics. Heterogeneity among studies was quantified using I2. When I^2^ > 40%, indicating a high and unacceptable risk of heterogeneity. A random-effects model was used for analysis, and sensitivity analysis or subgroup analysis was performed depending on the possible sources of heterogeneity.

## 3 Results

### 3.1 Literature search and screening results

**FIGURE 1:**
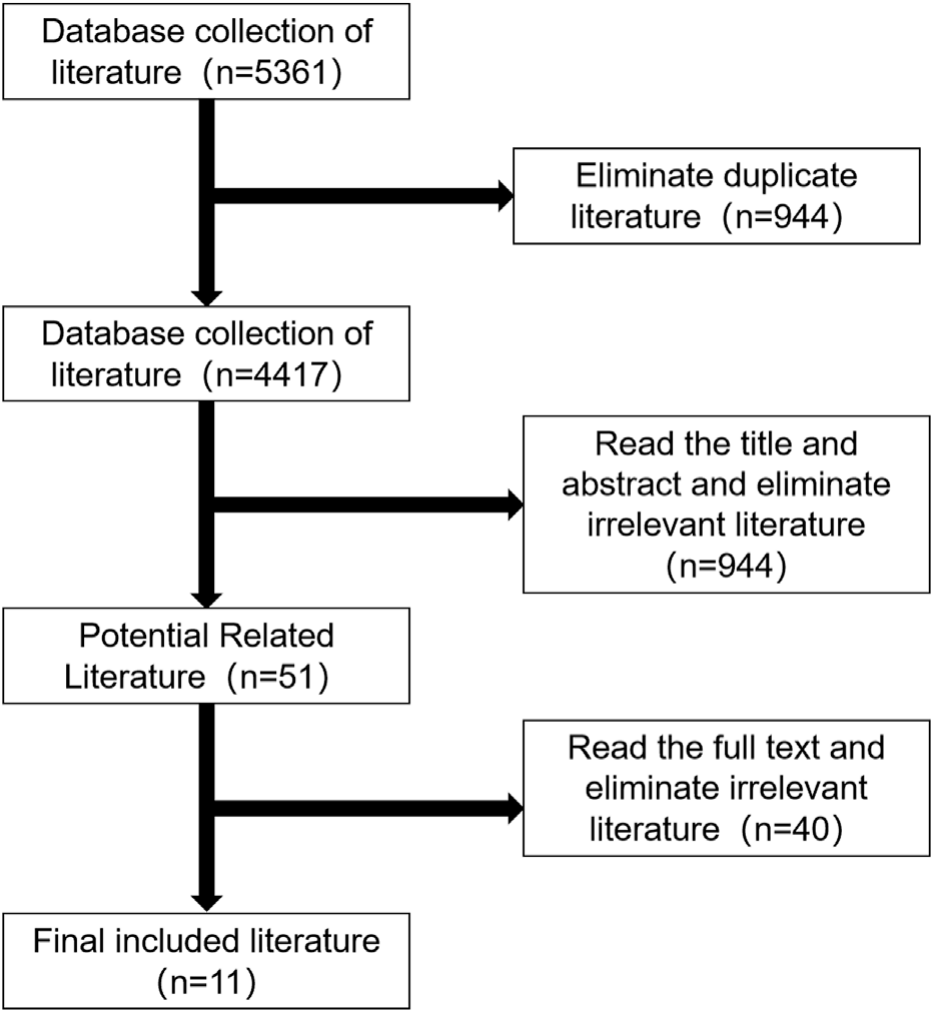
| Flow chart of literature screening.

The initial literature search yielded 5,361 articles, including 5,055 articles in English and 306 articles in Chinese. Using Endnote software to remove duplicate literature 944, read the title of the literature after the initial screening to obtain the literature 404, read the abstract after screening to obtain the literature 51, including the exclusion of nine articles cannot get the full text, the remaining 42 full-text reading to eliminate 31, and finally included 11 RCT, including eight English literature studies and three Chinese literature studies. The process of including the literature is shown in Figure 1.

### 3.2 Basic characteristics of the included studies and evaluation of methodological quality

Of the 12 included studies (Leehey et al., 2009; Straznicky, Lambert, Mcgrane, Dawood, & Schlaich, 2010; Toyama, Sugiyama, Oka, Sumida, & Ogawa, 2010; Aoike et al., 2014; Shi, Wen, Liu, & Yao, 2014; Craenenbroeck et al., 2015; Feng, Min, & Zun, 2016; Afifi et al., 2016; Fang, Zhigang, & Lei, 2017; Miele et al., 2017; Feng et al., 2018), a total of 410 subjects were included, and the basic information of the included studies is shown in Table 1.

**TABLE 1:** Basic information table of included literature.

Inclusion of studies and years	Age (years)	Gender	Sample characteristics	Stage of CKD	Sample size		Duration (weeks)	Type	Frequency (times/w)	Time	Intensity	Indicators^&^
					Control group	Exercise group						
Leehey et al. (2009)	Average age is 66	All male	CKD patients; obesity; diabetes mellitus; persistent proteinuria.	2-4	4	7	24	6 weeks (supervised walking on a treadmill), followed by 18 weeks of home exercise.	3	30–40 min	25–84% VO2 peak	1, 2, 3, and 4
Leehey et al. (2009)	Average age is 66	All male	CKD patients; obesity; diabetes mellitus; persistent proteinuria.	2-4	4	7	6	Walking	3	30–40 min	25–84% VO2 peak	1, 2, 3, and4
Straznicky et al. 2010	55±1	No mention	MetS* patients; nonsmoking; men and postmenopausal women	1-2	13	13	12	Cycling	Once in two days	40 min	65% HRmax	1, 2, and 3
Toyama et al. (2010)	71.7±11.0	Male: 89%	CKD patients with CVD**	3	9	10	12		Cycling (once a week) ＋ walking (every day)	30 min	Borg RPE grade 12–13	1
Shi et al. (2014)	69.4±7.7	Male/female = 14/7	CKD patients with CVD	3	10	11	12	Tai chi	3–5	30 min	—	1, 2, and 4
Liang et al. 2016	Average age is 48	Male/female = 30/28	CKD patients	2-3	29	29	12	Cycling	3	30 min	50% VO2 peak	1, 2, 3, and 4
Liang et al. (2018)	Male: 49.2±6.3; Female: 47.5±5.6	Male/female = 21/19	CKD patients	2-3	20	20	12	Cycling	3	30 min	50% VO2 peak	1, 2
Zhou et al. (2017)	Average age is 51	Male/female = 37/33	CKD patients	2-3	35	35	12	Cycling	3	30 min	50% VO2 peak	2 and 3
Miele et al. (2017)	35–70	No mention	CKD patients	3	21	25	16	Cycling	3	55 min	50-60% VO2peak	1
Craenenbroeck et al. (2015)	Average age is 51	Male/female = 22/18	CKD patients	3-4	21	19	12	Cycling	Every day	40 min	90% of heart rate at anaerobic threshold	1
Afifi et al. (2016)	45–55	Male/female = 29/21	CKD patients	3-4	20	30	12	Walking	3	15–20 min	Borg RPE grade 14	1, 2, and 4
Aoike et al. (2014)	Average age is 55	Male/female = 19/10	CKD patients	3-4	15	14	12	Walking	3	30 min	40–60% VO2max	1, 2, and 3

*MetS, metabolic syndrome; ** CVD: cardiovascular disease; ^&^indicator: 1 - eGFR, 2 - SCr, 3 - 24UP, and 4 - BUN.

**FIGURE 2:**
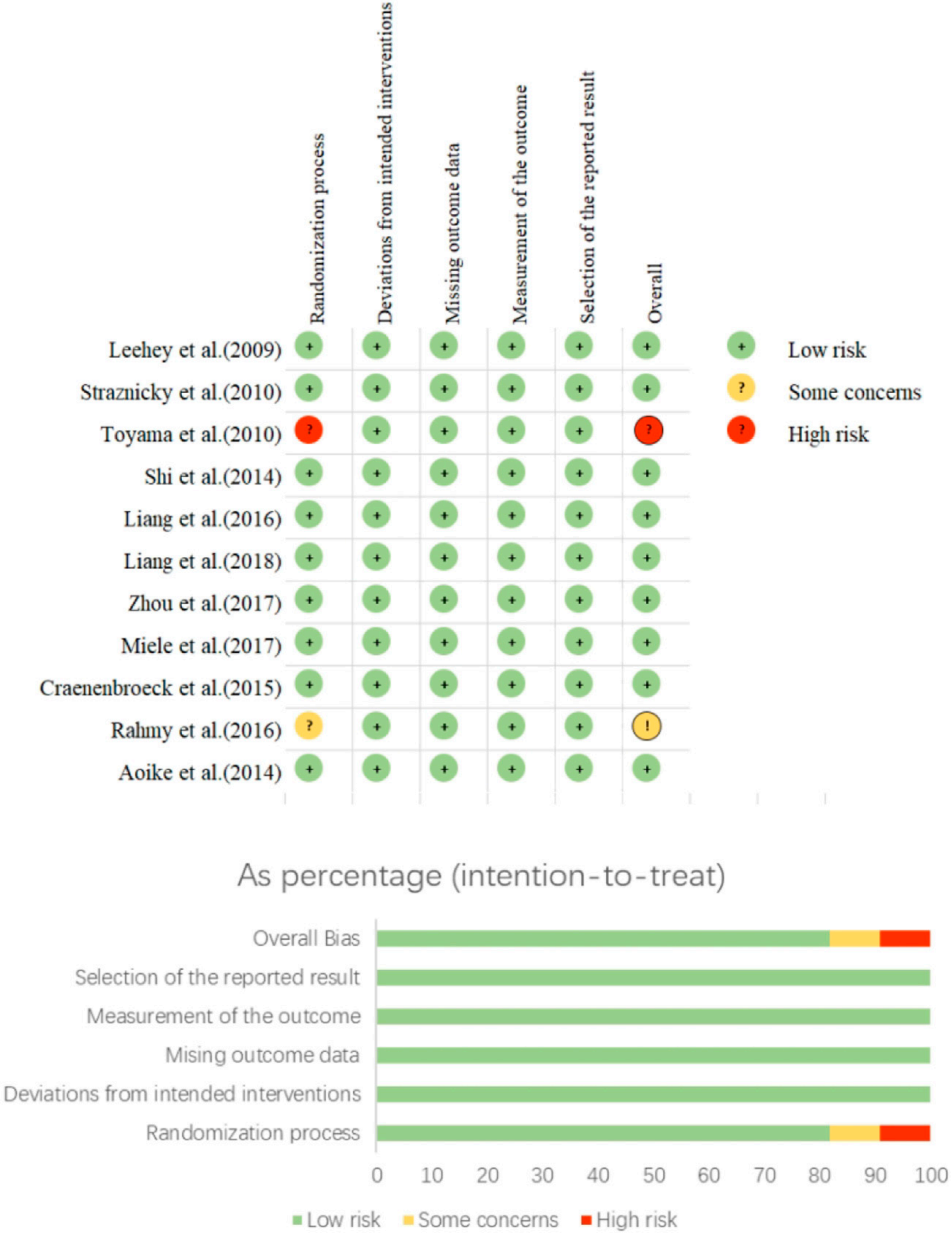
Risk bias diagram.

Nine of the 11 included articles were at low risk for the randomized design of the experiments, indicating more reasonable randomization in the grouping process. One article did not mention randomized grouping, and two others were uncertain; 12 studies were free of bias in both full presentation and selective reporting of outcome indicators, as detailed in Figure 2.

### 3.3 Publication bias test

**FIGURE 3:**
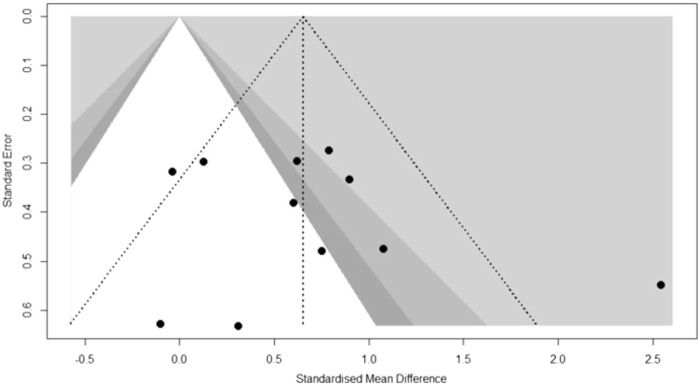
eGFR publication bias graph.

Funnel plots initially scatter plots using the treatment effect estimates for each study as the *x*-axis and the sample size as the *y*-axis to observe publication bias in articles. Funnel plots are not suitable if the literature is small, and publication bias is usually performed when the number of studies in the meta-analysis is 10 or more. In this study, there were 11 studies reporting eGFR, which could be tested for publication bias. From Figure 3, we know that the distribution of funnel plots about eGFR on the *x*-axis is symmetrically distributed on the left and right, indicating that there is no significant publication bias.

## 4 Meta-analysis results

### 4.1 Effect of aerobic exercise on eGFR in patients with CKD

**FIGURE 4:**
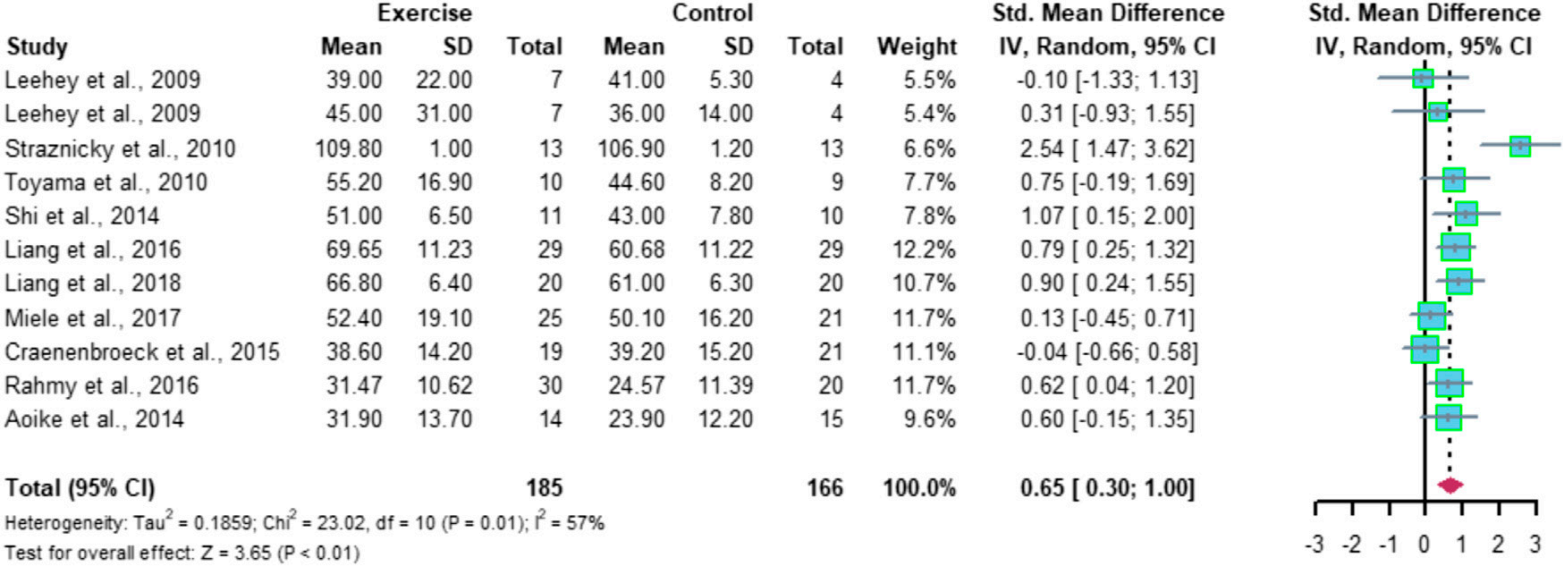
Meta-analysis of the effect of aerobic exercise on eGFR.

Eleven studies included the eGFR index with a total of 340 subjects (178 in the exercise group and 162 in the control group). Meta-analysis results showed high heterogeneity among the 11 studies (I^2^ = 57%, *p* = 0.01), using a random-effects model with a combined effect size of SMD = 0.65 and a 95% CI of [0.30, 1.00] (*p* < 0.01), indicating a significant difference between the two groups in terms of eGFR (Figure 4).

**FIGURE 5:**
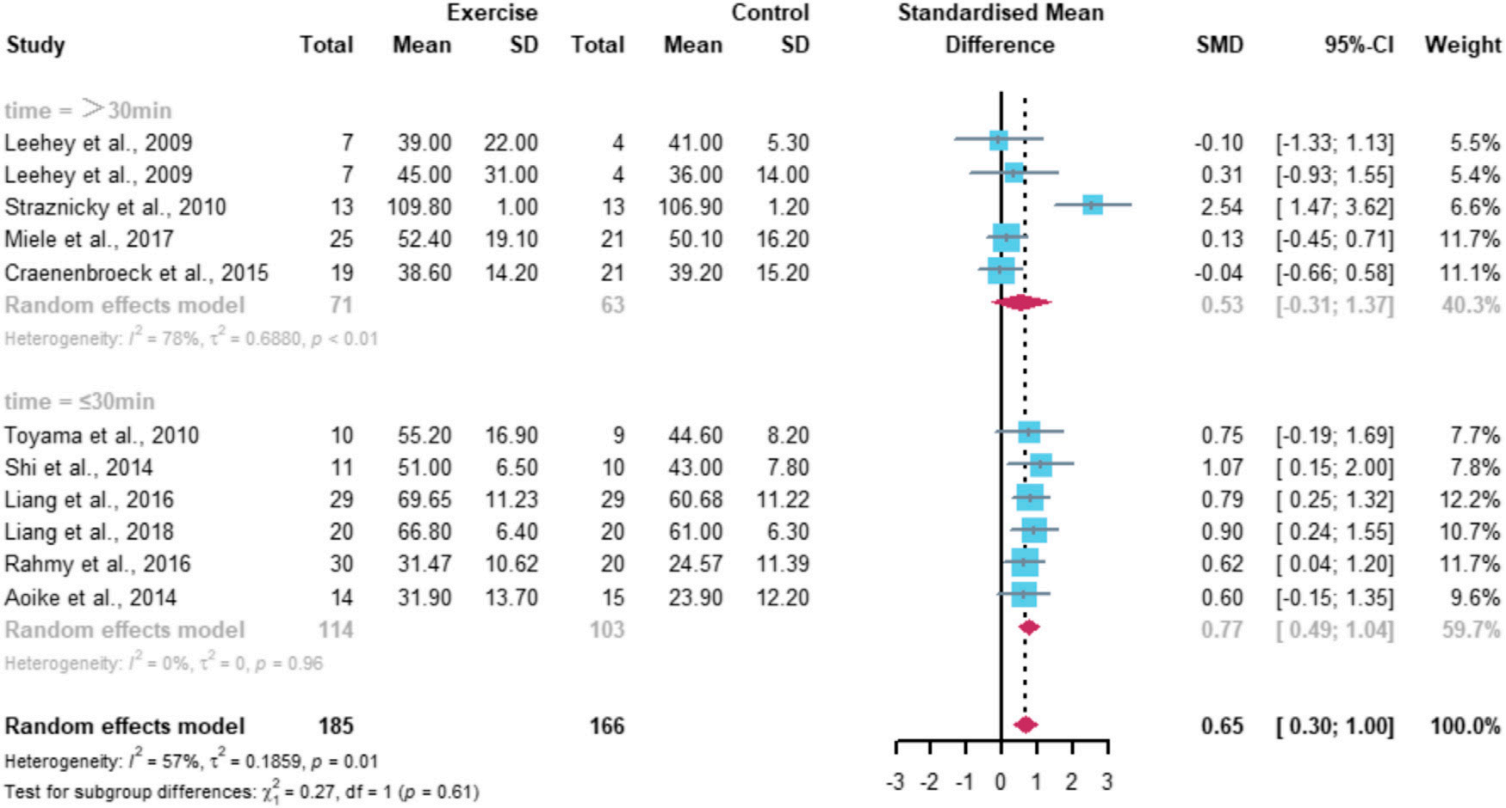
Subgroup analysis of the effect of aerobic exercise on eGFR.

Subgroup analysis was performed according to the sources that may cause heterogeneity (Figure 5). All studies were grouped according to the intervention duration of each intervention, with a total of five studies included in the subgroup of >30 min per intervention and six studies included in the subgroup of ≤30 min. The results showed that exercise with an intervention duration >30 min significantly improved eGFR (*p* < 0.01), while exercise with an intervention duration ≤ 30 min indicated that there was no significant difference between the exercise group and the control group (*p* = 0.96).

**FIGURE 6:**
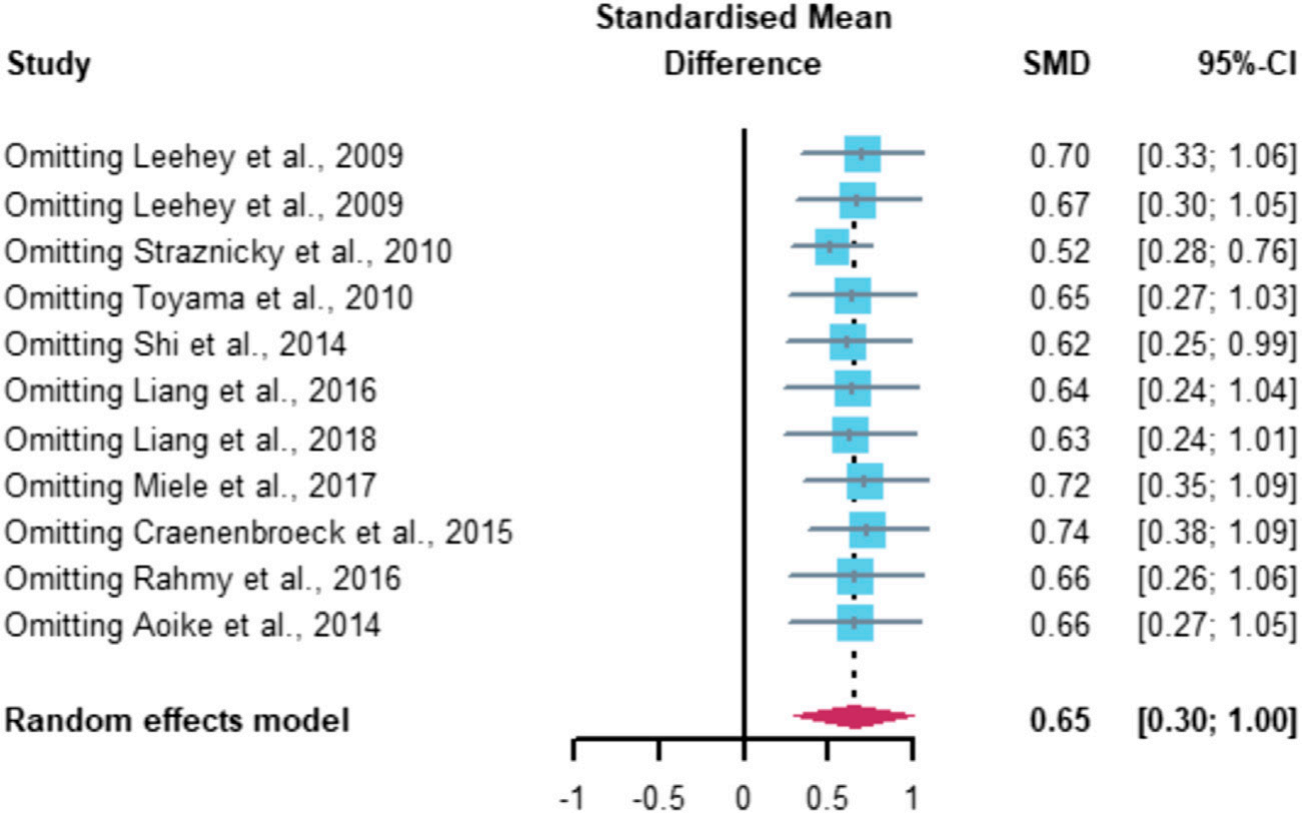
Sensitivity analysis of aerobic exercise affecting eGFR.

Sensitivity analysis was performed using a literature-by-literature exclusion approach (Figure 6) to find sources of heterogeneity, and it was found that the exclusion of any one literature did not result in a significant decrease in heterogeneity, indicating that the results of this part of the meta-analysis were more robust.

### 4.2 Effect of aerobic exercise on SCr in patients with CKD

**FIGURE 7:**
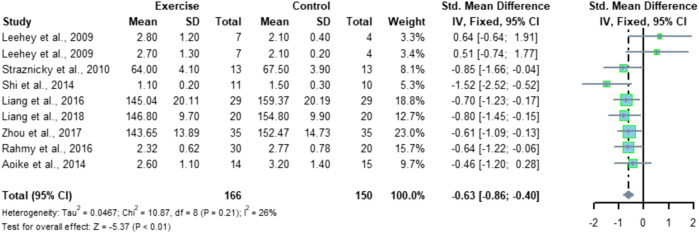
Meta-analysis of aerobic exercise affecting SCr.

Nine studies included SCr indicators with a total of 316 subjects (166 in the exercise group and 150 in the control group). Meta-analysis showed low heterogeneity between the nine studies (I^2^ = 26%, *p* = 0.21), using a fixed-effects model with a combined effect size of SMD = -0.63 and a 95% CI of [-0.86, -0.40] (*p* < 0.01), indicating a significant difference in SCr between the two groups (Figure 7).

**FIGURE 8:**
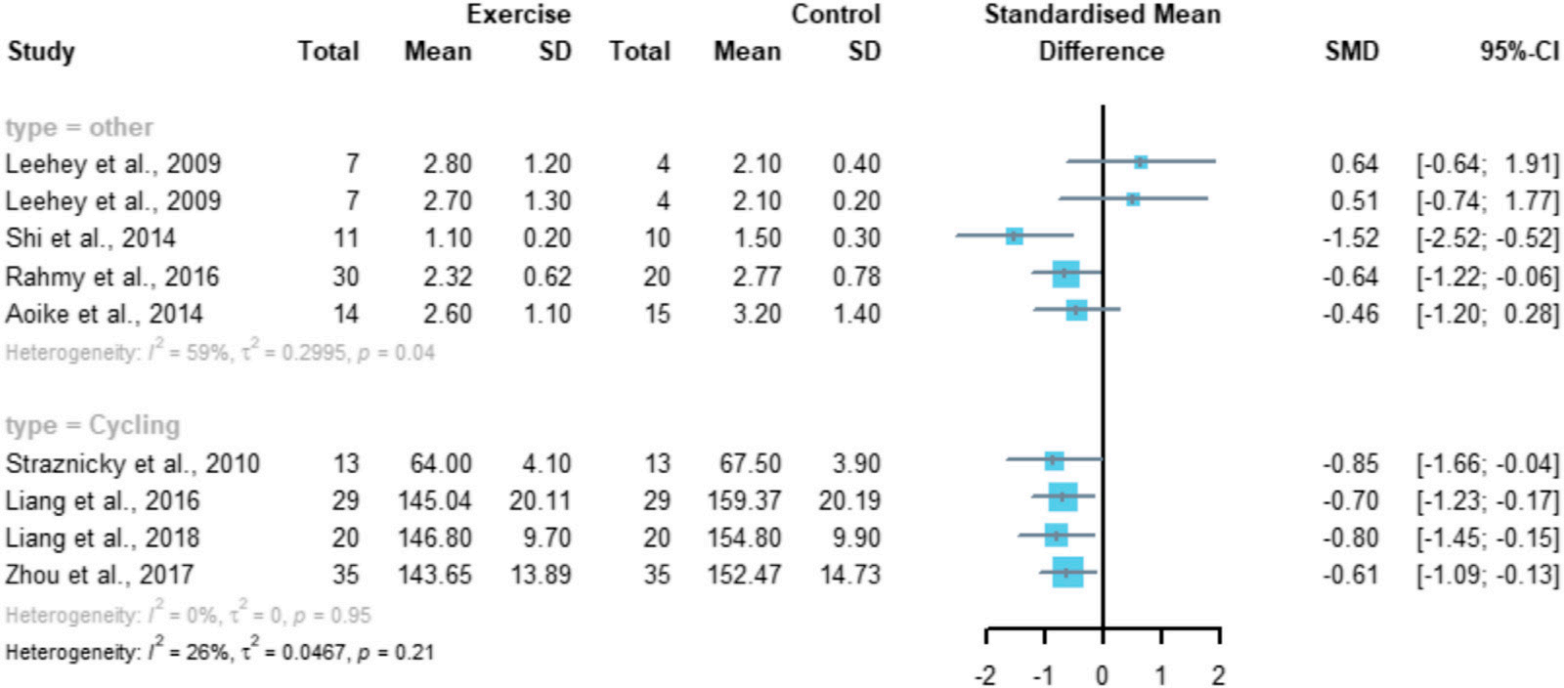
Subgroup analysis of the effect of aerobic exercise on SCr.

A subgroup analysis of factors that may affect the experimental results (Figure 8) was performed, and the grouping was based on the exercise modality, with a total of four studies using cycling as the exercise modality and a total of five studies included in the subgroup of other exercise modalities. The results showed that there was no significant difference in SCr between the two groups at the intervention with cycling as the exercise modality (*p* = 0.95), while performing other modalities of exercise such as walking and running was able to significantly improve the SCr levels in CKD patients (*p* < 0.05).

### 4.3 Effect of aerobic exercise on 24UP in patients with CKD

**FIGURE 9:**
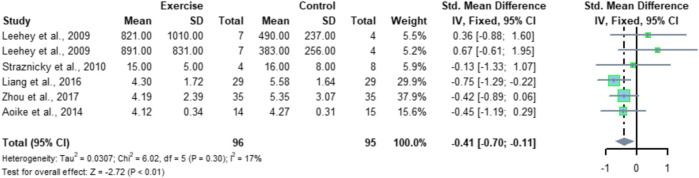
Meta-analysis of aerobic exercise affecting 24UP.

Six studies included 24UP indicators with a total of 191 subjects (96 in the exercise group and 95 in the control group). Meta-analysis showed low heterogeneity among the six studies (I^2^ = 17%, *p* = 0.30), using a fixed-effects model with a combined effect size of SMD = -0.41 and a 95% CI of [-0.70, -0.11] (*p* < 0.01), suggesting that exercise significantly improved 24UP (Figure 9).

### 4.4 Effect of aerobic exercise on BUN in patients with CKD

**FIGURE 10:**
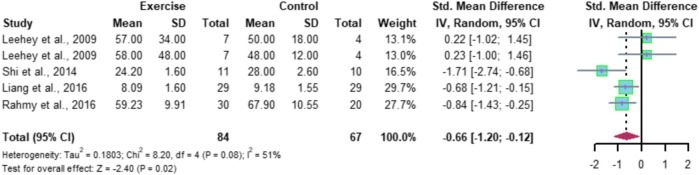
Meta-analysis of aerobic exercise affecting BUN.

Five studies included BUN indicators with a total of 151 subjects (84 in the exercise group and 67 in the control group), and the results of meta-analysis showed high heterogeneity among the five studies (I^2^ = 51%, *p* = 0.08), using a random-effects model with a combined effect size of SMD = -0.66 and a 95% CI of [-1.20, -0.12] (*p* < 0.05), indicating that aerobic exercise significantly improved BUN levels in patients with CKD (Figure 10).

**FIGURE 11:**
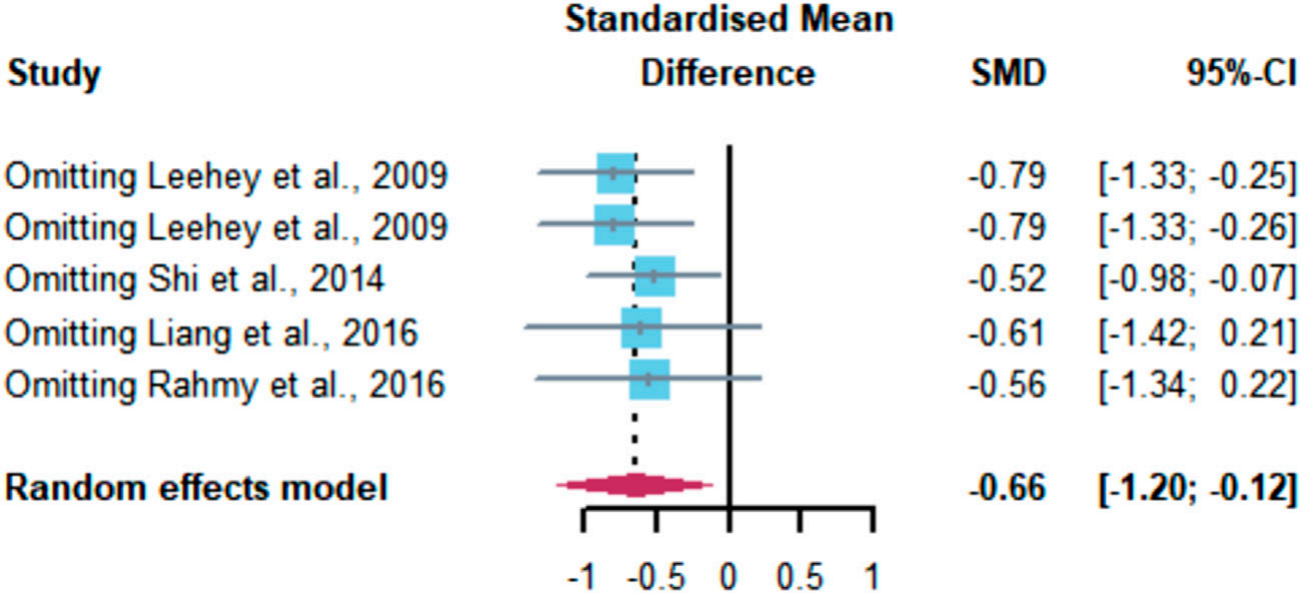
Meta-analysis of aerobic exercise affecting BUN.

Sensitivity analysis was performed using a literature-by-literature exclusion (Figure 11) to find the source of heterogeneity, and it was found that the heterogeneity was significantly lower after excluding the study by Shi et al. (2014) (I2 = 27.4%, *p* = 0.0599) with a 95% CI of [-0.98 -0.07] (*p* = 0.0241), indicating that the heterogeneity originated from the study by Shi et al. (2014).

## 5 Analysis and discussion

In recent years, the proportion of CKD patients in the elderly population has been increasing, and the global incidence is also increasing year by year. The current interventions for CKD are mostly achieved through dialysis treatment or surgery, which are expensive and have large side effects on the body. The quality of life is greatly reduced. With the development of the concept of “exercise is medicine,” the research direction of intervening in the process of chronic diseases through exercise has gradually come into public view (Barcellos, Santos, Umpierre, Bohlke, & Hallal, 2015). Meta-analyses exploring the improvement of the health status of CKD patients through physical exercise have also gradually emerged, but most of the current studies on the effects of exercise intervention in CKD patients have focused on exploring the positive effects of physical exercise on the locomotor capacity and some basic physical functions of the body (Heiwe & Jacobson, 2014; Clarkson, Bennett, Fraser, & Warmington, 2019; Villanego et al., 2020), while systematic evaluation studies directly analyzing the effects of exercise on renal function in CKD patients are still lacking. Therefore, the present study will focus on the effects of exercise on renal health.

Aerobic exercise is a recognized form of exercise that can improve the health of the organism, and the effects on the kidney have received widespread attention. Aerobic exercise ultimately has an impact on kidney function, mainly through the improvement of related proteins and kidney structure (Wang & Liu, 2009). It has been shown that aerobic exercise improves renal oxidative stress levels and enhances the renal antioxidant capacity. The adaptive changes in renal tissues are most pronounced at moderate intensity (Liu et al., 1999); it also improves body nitrogen reserves and serum protein levels, reduces body lipids, and avoids damage to renal filtration function from lipid peroxidation (Chen et al., 2000). In addition, aerobic exercise may inhibit renal fibrosis and improve renal structure by modulating the TGF-β1/Smad signaling pathway, thus improving renal function (Peng et al., 2021). As a chronic disease that endangers public health, whether aerobic exercise can play a role in the prevention and control of the disease course is a research direction worthy of attention. A 10-year follow-up study by Pechter et al. (Pechter, Raag, & Ots-Rosenberg, 2014) showed that the incidence of CKD was significantly lower in people who performed long-term aerobic exercise than in sedentary people. However, it is uncertain whether it can play a role in improving the renal function of CKD patients and whether factors such as the amount of exercise performed, the duration of exercise, and the basic condition of the subjects at the time of exercise can have an impact on the intervention effect. This study aimed to verify the effect of aerobic exercise on renal function in CKD patients. Meta-analysis of the included literature revealed that aerobic exercise had a significant improvement in estimated glomerular filtration rate, serum creatinine, 24-h urine protein, and blood urea nitrogen in CKD patients compared with controls (*p* < 0.05). Aerobic exercise with a single exercise duration >30 min had a significant improvement in eGFR (*p* < 0.01). Cycling as a means of aerobic exercise did not have a significant effect on the improvement of SCr in CKD patients (*p* > 0.05), while performing other forms of exercise such as walking and running could significantly improve the SCr level in CKD patients (*p* < 0.05).

### 5.1 Effect of aerobic exercise on eGFR

The amount of filtrate produced by both kidneys per unit of time is called glomerular filtration rate (GFR). The normal range for adult males is 125 ± 15 ml/min, and the normal value for adult females is about 10% lower than that for males. eGFR is mostly used in practical applications to estimate the kidney rate situation by estimating the index of glomerular filtration rate (eGFR). eGFR, as a traditional biological marker (Ntrinias et al., 2019), which can visually reflect the level of renal function, can be used in CKD to measure the degree of renal function and loss of functional renal units.

Meta-analysis of this study showed that aerobic exercise was able to have a significant positive effect on eGFR in CKD patients, SMD = 0.65, 95% CI [0.30, 1.00] (*p* < 0.01). After further subgroup analysis, the results showed that aerobic exercise of longer than 30 min per exercise session was required to significantly improve eGFR (*p* < 0.01), and exercise less than or equal to 30 min had no significant effect on this index, which indicates that aerobic exercise of longer single duration has a better effect on the improvement of eGFR in CKD patients. The study by Vanden Wyngaert et al. (2018) also presented similar findings, which may be related to the choice of aerobic exercise intensity. The study subjects in the literature included in this study were all CKD patients. Considering the subjects’ own conditions, the choice of the exercise intensities were all relatively small. Most of the exercise methods used were walking and cycling, which were less stimulating to the organism, so a longer exercise duration was required to achieve an effective exercise volume. From this, it can be inferred that aerobic exercise with a single duration of 30 min or more may play a positive intervention role in the disease process of CKD patients, but the selection and development of an effective specific exercise program still need further exploration.

### 5.2 Effect of aerobic exercise on blood renal function indexes

In this study, the blood indicators reflecting renal function were serum creatinine (SCr) and blood urea nitrogen (BUN). SCr is one of the most common methods to detect renal function in clinical practice and is an important indicator of renal function. Although it has been suggested that the diagnostic and prognostic significance of SCr and BUN as biomarkers of kidney diseases in the whole disease process is not ideal and cannot be used as the gold standard (Waikar, Betensky, & Bonventre, 2009), no new biomarkers have received wide public recognition yet (Wasung, Chawla, & Madero, 2015). Therefore, these two blood indicators reflecting renal function are still of great research significance.

Meta-analysis conducted in this study for the SCr index showed that aerobic exercise significantly improved the SCr level in CKD patients, SMD = -0.63, 95% CI [-0.86, -0.40] (*p* < 0.01). After further subgroup analysis, the results showed that conducting an aerobic exercise with cycling as an exercise modality did not significantly improve the SCr levels of CKD patients (*p* = 0.95), while performing other forms of exercise such as walking and running could significantly improve the SCr levels of CKD patients (*p* < 0.05), which indicates that performing whole-body aerobic exercise may have a better effect on improving the SCr levels of CKD patients and that CKD patients should also pay attention to the choice of exercise modality when performing exercise interventions, combining its effectiveness and their own conditions to carry out the exercise. This is not consistent with the findings of Zhang et al. (2019) that exercise therapy did not have a significant improvement on SCr, which may be due to the fact that there were too many types of exercise modalities included in the study to conclude the effectiveness of a certain form of exercise, and the results of this study can be integrated to infer that aerobic exercise may be one of the effective forms of exercise to improve SCr levels in CKD patients.

Meta-analysis conducted in this study for the BUN index showed that aerobic exercise significantly improved BUN levels in CKD patients, SMD = -0.66, 95% CI [-1.20, -0.12] (*p* < 0.05), which is similar to the results of animal experiments conducted by Duan et al. (2021), where aerobic exercise effectively reduced BUN levels in spontaneously hypertensive rats. In conclusion, aerobic exercise can play an effective role in improving all the indicators of renal function in the blood of CKD patients.

### 5.3 Effect of aerobic exercise on urine protein

The 24-h urinary protein (24UP) is measured by collecting all urine for 24 h to determine the amount of protein in it and then calculating the total amount of protein in 24 h. The amount of protein in normal urine is minimal, while the amount of urinary protein increases significantly when suffering from kidney disease or performing certain strenuous exercises. This index can be more. It is a marker of kidney injury and a predictor of the disease process in CKD (Ene-Iordache et al., 2016).

Meta-analysis of this study showed that aerobic exercise significantly reduced 24UP levels in CKD patients, SMD = -0.41, 95% CI [-0.70, -0.11] (*p* < 0.01), which is similar to the findings of Afshinnia et al. (Afshinnia, Wilt, Duval, Esmaeili, & Ibrahim, 2010). Proteinuria in the obese population was also significantly reduced after exercise intervention. Similarly, Yang et al.’s (2020) systematic review results suggest that exercise training in adult CKD patients does not aggravate proteinuria, but it is unclear whether there is a positive effect and that low to moderate intensity exercise may reduce proteinuria. The reason for this conclusion may be that CKD patients are usually older, and the included studies mostly selected lower intensity and milder forms of exercise for intervention. The body reacts more violently during high-intensity exercise. The results of this study suggest that aerobic exercise is an effective way to improve 24UP in CKD patients, and different forms of aerobic exercise such as running, swimming, and walking can be selected for an exercise intervention in practical applications.

## 6 Conclusion

Regular aerobic exercise had a significant improvement on the estimated glomerular filtration rate, serum creatinine, 24-h urine protein amount, and blood urea nitrogen in CKD patients (*p* < 0.05) and effectively alleviated the decline of renal function in CKD patients. Aerobic exercise with a single exercise duration of more than 30 min had a more significant improvement on the estimated glomerular filtration rate compared with cycling, walking, or running. Carrying out aerobic exercise can more effectively improve serum creatinine in CKD patients.

### 7 Limitations and their analysis

This study included literature on the length of the intervention cycle, which was mostly about 12 weeks, so it was not possible to compare the difference between the improvement effect of short-term and long-term exercise. Therefore, the conclusion of the study only explored the effect of regular aerobic exercise habits on the renal function of CKD patients. The effect of lifelong exercise habits on renal function still needs to be further investigated. Due to the lack of research in this field, this study is limited in exploring exercise modalities, and it cannot compare the specific differences between tai chi, swimming, square dance, and other exercise modalities preferred by the elderly population so as to give specific recommendations on exercise modalities. Due to the different designs of exercise programs in each study, it is difficult to unify the comparison of exercise intensity and exercise volume, and the comparison can only be made through exercise frequency and cycle length. Most of the study subjects included in this study in the literature were patients with stage 3–4 CKD. There is a lack of literature on the selection of patients with mild disease as study subjects in this field, so more researchers’ attention and exploration are still needed in exploring the preventive aspects of aerobic exercise.

## Data Availability

The original contributions presented in the study are included in the article/Supplementary Material; further inquiries can be directed to the corresponding author.
